# The Anti-Inflammatory Effects of Resistance Training in Patients with Type 2 Diabetes: A Systematic Review and Meta-Analysis

**DOI:** 10.3390/biom15101417

**Published:** 2025-10-05

**Authors:** Nikolaos P. E. Kadoglou, Chrysostomos Georgiou, Nikolaos Balaskas, Chrystalla Panayiotou, Michail Vardas, Andreas Mitsis, Constantine N. Antonopoulos

**Affiliations:** 1 Medical School, University of Cyprus, Nicosia 2029, Cyprus; cgeorg20@gmail.com (C.G.); nbalaskas1@gmail.com (N.B.); christia.pan.cp@outlook.com (C.P.); vardas.mic@gmail.com (M.V.); 2 Department of Cardiology, General Hospital of Nicosia, Strovolos 2031, Cyprus; andymits7@gmail.com; 3 Department of Vascular Surgery, Attikon University Hospital, National and Kapodistrian University of Athens, 124 62 Athens, Greece; kostas.antonopoulos@gmail.com

**Keywords:** diabetes mellitus, resistance training, adiponectin, interleukin-6, TNF-α, CRP, glucose

## Abstract

Type 2 diabetes mellitus (T2DM) is associated with increased cardiovascular risk characterized by low-grade inflammation. The aim of this systematic review and meta-analysis was to assess the effects of resistance exercise training (RET) predominantly on cytokines, along with changes in glucose profile and body composition in T2DM patients. The present systematic review and meta-analysis was conducted utilizing PubMed, Web of Science, Embase, and the Cochrane Library databases from their inception up to July 2024 (PROSPERO; registration number CRD420251149352). We screened only for randomized controlled trials investigating the effects of systematic, supervised RET on C-reactive protein (CRP) and adipokines: adiponectin, interleukin 6 (IL-6), tumor necrosis factor-alpha (TNF-α), along with changes in anthropometric indices and glycemic control in adult T2DM patients. Pooled post-exercise weighted mean differences (WMDs) with 95% confidence intervals (CIs) were calculated for all outcomes of interest between exercise-treated patients and controls. Sixteen studies involving a total of 668 T2DM patients were retrieved from the databases for meta-analysis. We used the GRADE framework for assessing the certainty of evidence. Cochran Q-score (I^2^) was used to estimate heterogeneity among studies (level of significance *p* < 0.10) and risk of bias analysis was also performed. The cumulative results showed that post-RET inflammatory markers were lower in exercise-treated patients compared to controls regarding CRP (mg/L) (WMD: −0.63; 95%CIs: −1.05, −0.20; *p* < 0.001); adiponectin (μg/mL) (WMD: −0.94; 95%CIs: −1.49, −0.38; *p* < 0.001). The results from adiponectin are quite conflicting since they derived from only three studies, where one of them had the greater impact. In parallel, we noticed significant amelioration of fasting glucose and HbA1c (*p* < 0.001), while body weight remained unaltered. Our meta-analysis demonstrated non-significantly lower levels of IL-6 and TNF-α in RET vs. control group. RET can merely reduce the inflammatory burden in T2DM patients by ameliorating the circulating levels of CRP and adiponectin, while in the rest of the biomarkers, non-significant results were obtained. Hence, the overall clinical impact of those anti-inflammatory effects of RET needs to be determined.

## 1. Introduction

Type 2 diabetes mellitus (T2DM) is a multi-faceted metabolic disorder with increasing prevalence in western-world countries [1]. It is highly associated with increased risk of cardiovascular diseases (CVDs), adversely affecting the prognosis of T2DM patients [2]. Sedentary lifestyle is a major environmental risk factor for T2DM development. The introduction of exercise for metabolic control of T2DM patients has long been the cornerstone of therapy [3,4]. The benefits of any type of systematic exercise training (aerobic, resistance, or mixed) on glycemic and lipid control are well-known [4]. Most importantly, systematic exercise exerts atheroprotective mechanisms beyond the modification of traditional CVD risk factors, with potential favorable effect on clinical outcomes [5]. In T2DM, the low-grade inflammation is depicted by the elevation of the wide-range inflammatory marker C-reactive protein (CRP) [6]. The adipose-tissue derivatives, named adipokines, link adiposity and CVD, and their modification can be a target of pharmaceutical interventions [7]. Significant differences between patients with T2DM and non-diabetic individuals have been found in serum concentrations of adiponectin, interleukin-6 (IL-6), tumor necrosis factor-a (TNF-α) [8], and resistin [9]. Apart from adiponectin, which is mainly considered as an anti-inflammatory cytokine and increases insulin sensitivity, the rest of the aforementioned adipokines promote inflammation and enhance insulin resistance [10]. The significance of these inflammatory markers is their correlation with CVD risk and their potential utilization in clinical practice in preventing and treating CVD in patients with T2DM [11].

A small number of studies have investigated the impact of any type of systematic exercise training on serum levels of adipokines in patients with T2DM [12]. However, previous systematic reviews and meta-analyses were at risk of bias since they included a very limited number of studies, weakening the evidence [13], or they did not distinguish studies based on the type of implemented exercise [14]. Despite their drawbacks, the already conducted systematic reviews showed the positive effects RET has on adults with T2DM by improving the population’s inflammatory and metabolic profile [13,14].

The aim of the present systematic review and meta-analysis was to examine the changes in serum levels of CRP, adiponectin, resistin, IL-6, and TNF-α, along with changes in glucose profile and body composition in adult T2DM patients following resistance exercise training (RET), compared to age- and sex-matched adults with similar characteristics, but not engaged in any form of exercise.

## 2. Materials and Methods

This meta-analysis included already published articles until July 2024. Reporting followed the Preferred Reporting Items for Systematic Reviews and Meta-Analysis of Diagnostic Test Accuracy Studies (PRISMA-DTA) [15] to ensure adherence to the PRISMA guidelines. This meta-analysis has been registered in PROSPERO (registration number CRD420251149352). The study protocol can be found on the same registry.

### 2.1. Criteria for Considering Studies for This Review

In the eligible studies, the participants had to be older than 18 years with established T2DM already receiving pharmaceutical regimen. According to the inclusion criteria, we only selected randomized controlled trial (RCT) comparing exercise versus control group. The former group underwent a systematic, RET program for at least 8 weeks and at least 3 times per each week. All RET sessions were structured and performed under supervision in groups of patients or personal training. Those criteria of frequency, intensity, and duration were applied to ensure that exercise quantity achieved a sufficient level. The control group was matched to the intervention group, but it did not follow any exercise training program during the study. RCTs measuring at least one of the following blood inflammatory markers were included in meta-analysis: CRP, adiponectin, resistin, visfatin, vaspin, apelin, IL-6, and TNF-α. Some studies have reported the hsCRP, which depicts a more sensitive assay of CRP, especially in the low range of its values. However, that was not the case in our meta-analysis, since CRP in all studies was elevated and the concentrations were comparable between those two techniques. Our initial goal was to include all those adipokines in our meta-analysis. However, there was an inadequate number of RCTs (<3) for systematic review and meta-analysis measuring resistin, visfatin, vaspin, and apelin, so we did not proceed with the latter adipokines. We also excluded studies reported only in abstract form, uncontrolled reports (case series, case reports), literature reviews, systematic reviews, meta-analysis and studies with unavailable data. Studies including chronically ill T2DM patients with high inflammatory burden and poor prognosis (e.g., concomitant severe CKD, severe HF) were not eligible.

### 2.2. Search Methods

For studies’ selection we utilized both electronic platforms and physical resources. We searched for studies published in English from their inception up to July 2024. The electronic platforms we looked into were MEDLINE, EMBASE, Web of Science, and the Cochrane Library. In addition, we also searched regional electronic bibliographic databases, subject-specific databases, and dissertations. In our search we also included any published, unpublished, or ongoing studies and their relevant references that could be found in the Science Citation Index database. To find these studies we searched for titles and abstracts including the following terms: type 2 diabetes mellitus, RET, resistance exercise training CRP, adipokines, adiponectin, IL-6 and TNF-α, fasting plasma glucose (FPG), glycosylated hemoglobin (HbA1c), body weight, body-mass index (BMI), waist-hip ratio (WHR), fat mass. Finally, we searched already published meta-analyses and literature reviews for additional references.

### 2.3. Data Collection and Extraction

To ensure that all included studies were indeed suitable for our meta-analysis, two independent researchers double assessed them using the abovementioned criteria. Firstly, the studies were assessed based on their title and abstract, and if deemed possibly suitable, the full text was read to reach the final decision on whether the study will be included or not. A disagreement between two researchers was resolved after discussion and consulting a third-party author. Data extraction was performed independently and in duplicate by two researchers using a predefined electronic spreadsheet. In case of a disagreement between the two researchers in data extraction, a third experienced researcher decided for the specific study.

### 2.4. Assessment of Methodological Quality

Similarly to data collection and extraction, the assessment of methodological quality was also performed independently by two independent researchers. The QUADAS-2 tool, a revised version of the original QUADAS tool, was used for the assessment of the selected studies. Additionally, the public user-testing version of the QUADAS-2c tool was used to compare the index tests. We used the GRADE framework for assessing the certainty of evidence. If the above tools were not of sufficient effectiveness, the authors were contacted and asked to provide clarifications and additional information to ensure the quality of all included studies.

### 2.5. Statistical Analysis and Data Synthesis

**Figure 1:**
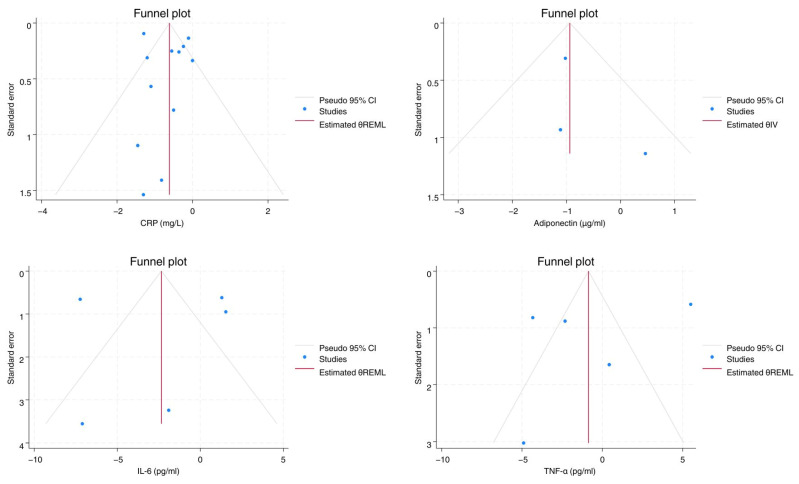

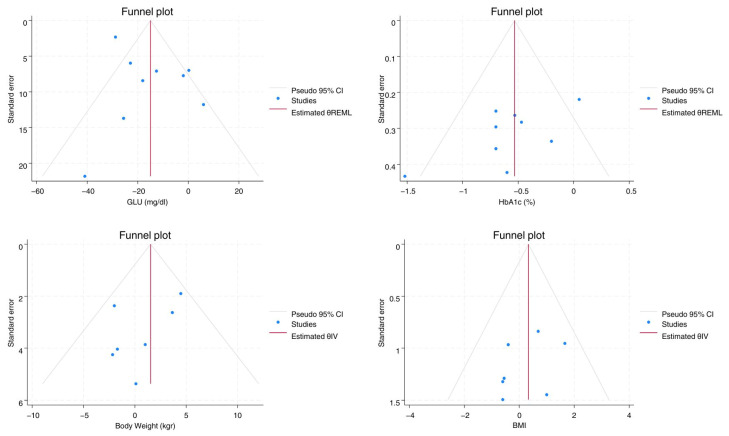
Funnel plots of examined parameters.

The meta-analysis was performed according to the Cochrane Handbook for Systematic Reviews of Interventions version 6.2 (updated February 2021) [16]. A RCT is to compare the effects between the intervention group and the control group after the end of the trial period. All selected studies included in this meta-analysis did measure the post-RET weighted mean differences (WMDs) of all the data included in this study between the two groups using means, standard deviations (SDs), and sample sizes. Thereafter, the pooled estimate of the WMDs with 95% confidence interval (CIs) was calculated for each outcome by pooling the WMDs from the respective studies. We used the Cochran Q-score (reported as I2) to estimate heterogeneity among studies and level of significance was set at *p* < 0.10. It was not feasible to conduct sensitivity or subgroup analysis to explore the sources of heterogeneity due to the small number of included studies. Meta-analysis was performed using random-effects, DerSimonian-Laird model; and fixed-effects, inverse-variance model. For the assessment of publication bias we used the visual inspection of the funnel plots (Figure 1), along with Egger’s regression coefficient (Table 1). The meta-analyses were performed with STATA v17.0, (STATA Corp., College Station, TX, USA).

## 3. Results

**Figure 2:**
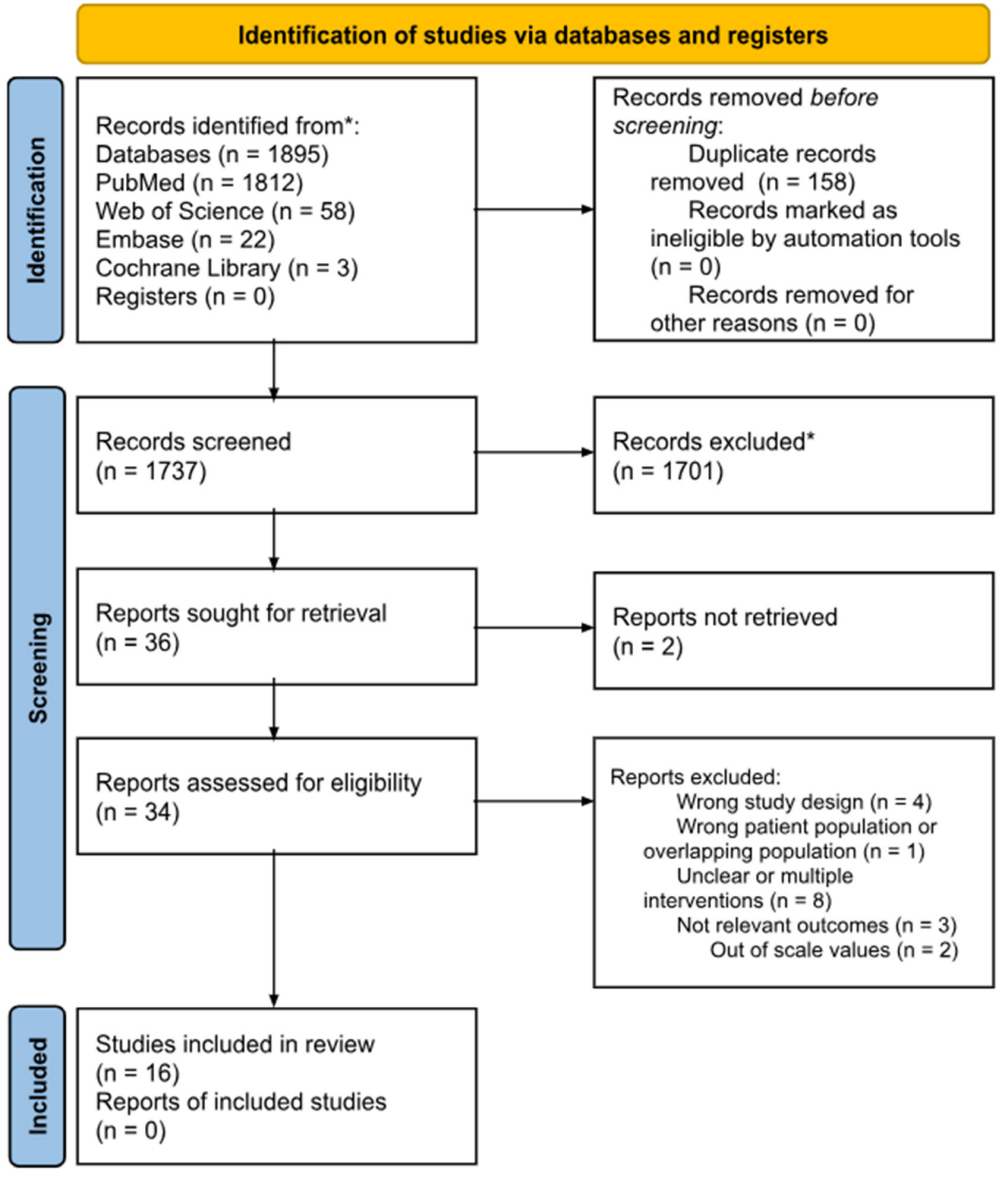
PRISMA flow diagram of studies selection in the systematic review and meta-analysis. * Records excluded based on title and abstract.

After the exclusion of 1895 studies, a total of 16 studies were included in the present meta-analysis of end results. All analyzed parameters were valued, except WHR and the aforementioned adipokines where the collected RCTs were less than three for each of them and a meta-analysis could lead to unsafe conclusions. The characteristics of all included studies are summarized in Figure 2 and Table 2.

**Figure 3:**
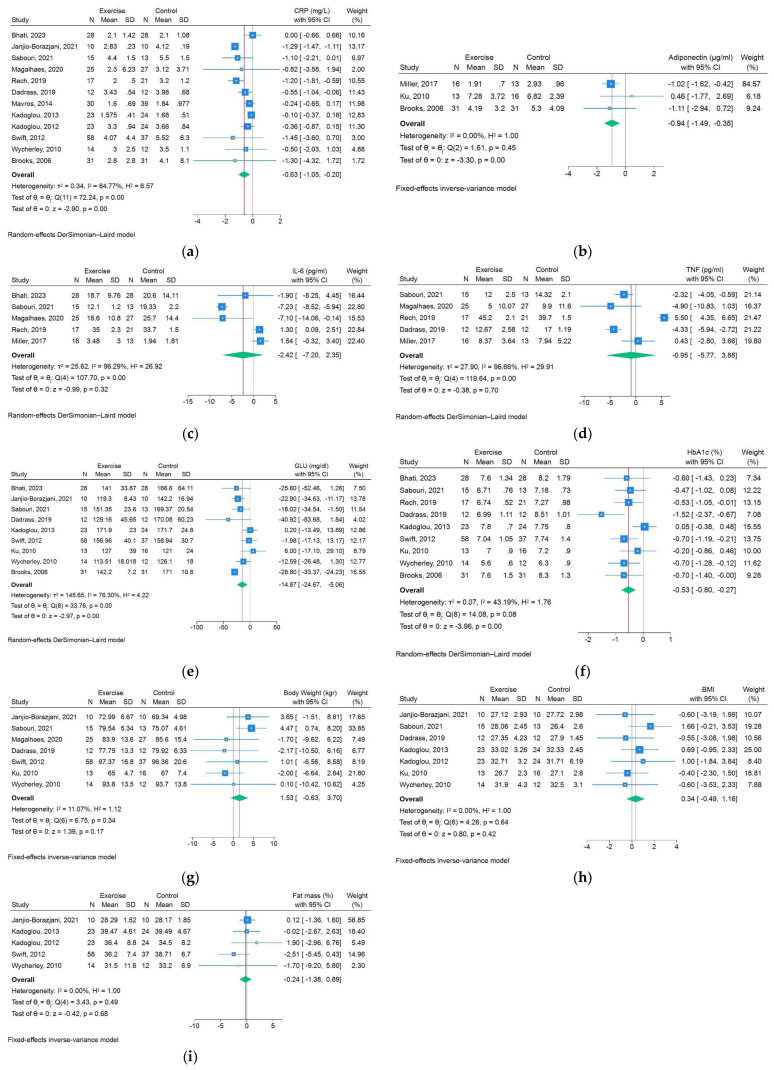
Forest plot of post-intervention values’ comparison between exercise and control groups for (**a**) CRP; (**b**) adiponectin; (**c**) IL-6; (**d**) TNF-α; (**e**) fasting plasma glucose; (**f**) HbA1c; (**g**) body weight; (**h**) BMI; (**i**) fat mass [5,17,18,19,20,21,22,23,24,25,26,27,28,29,30,31].

**CRP:** A total of eleven studies were found to calculate the alterations in CRP levels in control and RET groups of patients with T2DM. A significant reduction in CRP levels was noted in the RET group, in comparison to the control group (WMD: −0.63 mg/dL; 95%CIs: −1.05, −0.20; *p*-value < 0.001) (Figure 3a).

**Adiponectin:** A total of three studies were found to calculate the alterations in circulating adiponectin levels in control and RET groups of T2DM patients. Significant increase in adiponectin levels was noted in the RET groups compared to the control groups (WMD: −0.94; 95%CIs: −1.49, −0.38; *p*-value < 0.001). However, those results were driven by Miller’s study, while the other two studies showed the opposite effects of RET, raising concerns about its generalizability (Figure 3b).

**IL-6:** After calculating the levels of IL-6 at the end of each study, we demonstrated considerably lower IL-6 levels after RET compared to controls (WMD: −2.42; 95%CIs: −7.20, 2.35; *p*-value = 0.32). We decided not to include Dadrass et al.’s study as a significant outlier (Figure 3c).

**TNF-α:** The impact of RET on TNF-α blood levels seemed controversial, since four out of seven RCTs reported lower TNF-α levels in RET-treated patients than controls, while the remaining three RCTs showed the opposite results. In the end, the difference in TNF-α levels between groups did not achieve a significant level (WMD: −0.95; 95%CIs: −5.77, 3.88; *p*-value = 0.70) (Figure 3d).

**Resistin:** We found only two studies fulfilling our selection criteria, without any significant difference between RET and control groups. We did not proceed to meta-analysis.

**Glycemic profile:** The levels of circulating fasting plasma glucose (FPG) and glycosylated hemoglobin (HbA1c) were also studied in the selected RCTs. RET yielded in significant improvement of both glycemic indices compared to control group (FPG; WMD: −14.87; 95%CIs: −24.67, −5.06; *p*-value < 0.001; HbA1c; WMD: −0.53; 95%CIs: −0.80, −0.27; *p*-value < 0.001) (Figure 3e,f).

**Body composition:** Among the selected RCTs, seven provided measurements of body weight, while eight studies of BMI in both groups of T2DM patients. A slightly higher body weight and BMI was revealed in the RET group in comparison to the control, without achieving the significance level, respectively (body weight; WMD: 1.53; 95%CIs: −0.63, 3.70; *p*-value = 0.17; BMI; WMD: 0.34; 95%CIs: −0.49, 1.16; *p*-value = 0.42) (Figure 3g,h). Regarding WHR, the existing two studies did not provide adequate evidence to draw conclusions.

**Fat mass:** A total of five studies included measurements of fat mass changes between the RET group and the control group. Out of these studies, none showed any significant differences between the intervention and the control group (WMD: −0.24; 95%CIs: −1.38, 0.89; *p*-value = 0.68) (Figure 3i).

**Risk of bias analysis**: A total of sixteen studies were included in this meta-analysis, the majority of which were at low risk of bias. Fifteen of the included studies clearly stated that randomization was implemented when creating the study’s groups and seven of those used allocation concealment to avoid bias from the research staff. Unfortunately, due to the nature of exercise intervention studies, blinding was difficult to be implemented. Interestingly, there was an exception with one study (Mavros 2014 [26]) being double-blind by using a sham-exercise group as controls. The rest of the studies were listed as high risk in this domain. Four studies worked with independent laboratories to obtain the values of the assessed variables and so they were listed as low risk in the outcome assessment domain. As it is expected in exercise intervention studies, the majority of them faced dropouts. The studies that clearly explained the dropout reasons and/or mentioned following an intention-to-treat analysis to overcome this common limitation were listed as low risk in the incomplete outcome data domain (Table 3).

## 4. Discussion

This is the third published meta-analysis investigating the impact of systematic resistance training program on inflammatory markers. Sixteen randomized control trials (*n* = 668) were included in this meta-analysis with sample sizes of the exercise group ranging from *n* = 6 to *n* = 31. We observed a modest, but statistically significant, decrease in circulating levels of CRP and adiponectin, while the rest of the inflammatory markers, namely TNF-α and IL-6, showed a trend for lower concentrations, but that difference did not achieve a statistically significant level. The overall anti-inflammatory benefit as observed in the present meta-analysis is not robust and requires further investigation.

Previous RCTs have shown a wide variance of RET-related effects on metabolic and other parameters. Differences in their essential characteristics: type of exercise, duration and intensity, and the disease status, as well (e.g., existent complications), may provide a plausible explanation. Besides this, a few studies have tested the impact of combined exercise and dietary interventions [32,33]. In our meta-analysis, we tried to limit confounders and focus on the net anti-inflammatory effects of RET. Compared to previously conducted meta-analyses, we confined our search strategy among RCTs applying only RET, with specific minimum characteristics, to ensure adequate efficacy. Thereby, we excluded heterogeneous RCTs with mixed types of exercise interventions. Unfortunately, other potential confounders like medications or diet were not available, so our analysis was not adjusted for them. Our statistical analysis complied with current instructions from the Cochrane database. A strength of our systematic review and meta-analysis was the parallel assessment of glycemic control and body composition parameters to assess pathophysiological mechanisms. In agreement to previous studies and meta-analyses [34,35], we confirmed the improvement of glycemic profile (both FPG and HbA1c) after RET. Despite the glucose lowering, the body weight, BMI, and fat mass did not differ between exercise and control groups, while body weight tended to increase in the exercise group. Our findings implicate the favorable effect of RET on glycemic profile, independent of body composition changes. A plausible mechanism can be the amelioration of insulin sensitivity, leading to increased glucose absorption by skeletal muscles through the more intensive muscular training by RET [36,37,38]. Unfortunately, we did not have adequate data to examine this hypothesis.

**CRP:** It is an acute phase protein produced by the liver and adipocytes that plays an important role in signaling between immune system cells and, concomitantly, is a widely accepted index of systemic inflammation [39]. Most importantly, CRP has been already established in clinical practice as an essential part of the diagnostic and therapeutic work-up of many chronic diseases. In our study, we observed a moderate but significant reduction in circulating CRP levels, which is in line with previous meta-analyses [13,39]. With the exception of Bhati 2023 [17], which mentioned no difference between the intervention and the control group at the end, the CRP reduction was unanimously mentioned by almost all included studies in our meta-analysis. Although the exact mechanism leading to exercise-induced CRP reduction is not fully understood [34], it is speculated that exercise exerts anti-inflammatory action via the suppression of multiple factors involved in the pro-inflammatory pathways [17,39,40]. Regarding the positive association of CRP with cardiovascular disease, such as myocardial infarction and stroke, the lowering of its levels indicates a “pleiotropic”, anti-inflammatory effect of RET in this chronic systemic, low-grade inflammatory condition, with potential benefit in the future [41]. The amount of accumulated reduction was moderate and may not translate to meaningful cardiovascular risk amelioration. However, CRP lowering by RET highly supports the anti-inflammatory impact of RET.

**Adiponectin:** We demonstrated a statistically significant decrease in adiponectin levels in the intervention rather than control groups. Notably, in our analysis, that effect was mostly driven by one study (Miller et al. 2017) (weight 84.57%) [27]. Moreover, the number of included RCTs showed high heterogeneity. Those factors may dispute the final result from the methodology point of view. Previous studies and meta-analyses have documented conflicting results regarding the effect of exercise training on adiponectin [39,42]. In particular, they have shown either increased or decreased, or no changes, in the adiponectin levels following exercise intervention, compared to controls [39,42,43]. The above also applies to meta-analyses studying the effects of aerobic exercise on adiponectin, as this adipokine was found to be increased following intervention [43], but the results are still conflicting, with outliers present [12]. A plausible explanation may derive from the interplay between adiponectin and its receptors. Systematic RET may activate the adiponectin receptor 1 pathway, leading to higher adherence to the receptor and so lower circulating levels of adiponectin [44]. Moreover, adiponectin concentrations have been inversely associated with muscular performance in adolescents after adjustment for many demographic and metabolic factors [45]. The role of adiponectin in CVD development, especially among diabetic patients, has not yet been fully understood [46,47]. It is suggested that increased circulating adiponectin levels may act as a protective factor against CVD [48]. There is also evidence that adiponectin may not be actually involved in CVD development, but it is assumed as a bystander of several pathways dragged by the changes in other inflammatory markers [49]. It has also been suggested that elevated adiponectin levels may increase CVD incidence and mortality, a phenomenon referred to as the “adiponectin paradox” [47]. Besides this, we cannot rule out the bystander effect of RET on adiponectin without any mechanistic link. Thus, the clinical significance of our finding on adiponectin remains unclear and further research is required to fully unmask the controversial relationship of adiponectin and CVD to draw firm conclusions about the clinical significance of exercise-induced adiponectin changes [49]. Additionally, other studies have opted towards measuring adiponectin changes in relation with other adipokines, like leptin, showing fewer variable results in contrast to measuring circulating adiponectin levels alone. Hence, a cluster of adipokines could more realistically depict the inflammatory changes following exercise interventions [50].

**IL-6:** Interleukin-6 is a pleiotropic pro-inflammatory cytokine produced by a variety of cells, including B-cells, T-cells, and adipocytes [51]. It is also produced by myocytes, also known as myokine, and it is vital for immune responses and has been involved in the pathogenesis of multiple chronic inflammatory and autoimmune diseases [52]. Paradoxically, limited past evidence showed that IL-6 also has anti-inflammatory properties by taking part in metabolism regulation, cell regeneration, and bone homeostasis [53]. Our meta-analysis showed no difference in IL-6 levels between the intervention and control group at the end of the studies. This result is supported by previously conducted studies, showing either a decrease or no difference between RET and controls [54,55]. Interestingly, despite the reduction observed in CRP and our knowledge of the close relation of the two inflammatory markers, IL-6 did not follow the same pattern [56]. Perhaps, the regulation of IL-6 as a myokine might have confounded the impact of RET on its levels. Additionally, it has been found that IL-6 levels can even increase significantly following strenuous exercise [57]. The degree of this inflammatory cytokine’s elevation is closely related to the duration and intensity of the workout, as its levels are linked with the extensiveness of post-workout muscle damage [57]. However, prolonged and exaggerated local inflammatory response is avoided through the concomitant production of anti-inflammatory cytokines [57].

**TNF-α:** Tumor necrosis factor-alpha is an important cytokine used to assess systemic inflammation [58] that regulates multiple pro-inflammatory responses, increases oxidative stress, and takes part in chronic inflammation, obesity, and insulin resistance processes [59]. It plays a key role in several aspects of inflammatory cascade, controlling the action of many other cytokines (Kadoglou et al., 2012) [23]. Our meta-analysis showed no difference in TNF-α levels between the exercise and control groups. On the contrary, previous studies showed that RET can significantly lower circulating TNF-α concentration [54,60]. This observation is partly due to the inclusion of the Rech et al. 2019 study [28], because despite the relatively homogenous weight distribution of each study contributing to the TNF-α results in our meta-analysis, the study in reference was an outlier (Figure 2). TNF-α level changes can be a useful tool when assessing the severity and prognosis of chronic diseases, including CVD, as it is related to the development and progression of atherosclerotic plaques [58].

Overall, we should extrapolate the above results into everyday clinical practice. This meta-analysis included T2DM patients without CVD. The most common characteristics of implemented RET protocol for T2DM population is shown in Table 2 and included RET sessions three times a week, 50–60 min per session, at approximately 70% of one-repetition maximum. Our results showed that RET has the potential to ameliorate a few inflammatory factors, especially CRP, despite a non-significant change in body weight. On the other hand, the absence of significant impact of RT on other markers, like IL-6 and TNF-α, questioned an effective global anti-inflammatory effect of RT. We did not include diabetic patients with already established CVD, which could provide direct evidence of the beneficial outcomes of exercise training on CVD progression. When compared to aerobic exercise (G. Papagianni et al., 2023 study), the former exercise type seems superior in decreasing the studied inflammatory markers levels, namely CRP, TNF-α, IL-6, and resistin [12]. Aerobic exercise may also improve body composition, while the same result was not observed in the RET groups of our meta-analysis [12]. Notably, the improvement of glycaemic markers levels, namely HbA1c and FPG, seemed comparable between the two exercise types. However, RET, even though it provides some beneficial effects, does not significantly improve cardiorespiratory fitness, especially when compared to aerobic exercise, with the latter’s effects being more well documented [61]. Moreover, the small number of studies prevented us from obtaining any gender effects, which has been supposed in adipokines.

## 5. Conclusions

To summarize, RET in the context of a healthier lifestyle should be advised in the adult T2DM population to reduce the cardiovascular risk through the amelioration of multiple metabolic and, presumably, anti-inflammatory mechanisms. However, it is important to point out that the overall anti-inflammatory effect of this intervention is modest and primarily driven by the amelioration of CRP levels, a well-established inflammatory marker. In the rest of the adipokines, our meta-analysis showed a weak increase (adiponectin), mixed, or non-significant results among the limited number of heterogeneous studies.

## 6. Limitations

Our meta-analysis has several limitations. First, the total number of studies we included was relatively small, because of the lack of studies fulfilling the set criteria. In some parameters, like adiponectin, only three studies were available. In addition, some of these studies did not include all the data we needed, specifically the samples’ age and gender distribution. Secondly, while one of the strengths of our meta-analysis was the inclusion of RCTs only, an important limitation of our study was the highly increased risk of bias, as the included studies appeared to have high heterogeneity (I^2^ > 75%) and low quality, which could skew pooled estimates. Most interventional exercise studies usually apply different exercise protocols worldwide, making their comparison problematic and sometimes impossible. Moreover, all studies were unblinded, which is common and unavoidable in exercise studies, increasing the performance bias. Except for CRP, all the rest of the parameters were measured by ELISA from different manufacturers, unfortunately using their own control curves. Up to now, there are no validated cut-off values, and those parameters are assayed for research purposes. In addition to this, the primary studies selected for our meta-analysis did not provide data like smoking status, dietary habits, and medications. They consist of significant parameters of everyday clinical practice, which may affect the inflammatory and metabolic profile and, therefore, their changes after interventions [62]. We focused on the net effect of RET on circulating parameters, but the lack of such information around these variables could possibly limit generalizability of our results. Finally, a significant disadvantage of all exercise-intervention programs is the inability to obtain long-term data from studies like the ones included in our meta-analysis, as they usually do not last more than four months. This is due to the high drop rate of the diabetic population participating in intervention studies and the lack of funding available for such long-term projects. The absence of registration of long-term clinical outcomes limits translational conclusion. Any effects on morbidity and mortality have not been examined in the short-term studies, but they should be evaluated in long-term studies. The proposed anti-inflammatory mechanisms with potential cardiovascular benefits gained from a healthier lifestyle with systematic RET remain to be proved.

## Figures and Tables

**Table 1:** Summary of Egger’s tests.

Regression-Based Egger Test for Small-Study Effects	
Outcome	*p*-Value
CRP	0.53
Adiponectin	0.40
IL-6	0.60
TNF-α	0.44
GLU	0.90
HbA1c	0.03
Body weight	0.17
BMI	0.36
Fat mass	0.72

**Table 2:** Randomized control trials included in the meta-analysis studying the effects of systematic, resistance exercise training (RET) on inflammatory markers in patients with type 2 diabetes mellitus.

References	Country	Exercise Cohort (Sample Size, Gender (M/F), Age)	Control Cohort (Sample Size, Gender (M/F), Age)	Exercise Intervention (Frequency, Session Duration, Total Duration, Intensity)	Post-Intervention Inflammatory Variables in RET vs. Controls
Bhati 2023 [17]	India	28 (15/13) pts 52.8 ± 6.82 y	28 (17/11) pts 54.0 ± 8.18 y	3 sess/wk, 60 min/sess, 12 wk, 65–75% of 1RM	↔ hsCRP, ↓ IL-6
Brooks 2006 [18]	USA	31 (21/10) pts 66 ± 2 y	31 (19/12) pts 66 ± 1 y	3 sess/wk, 35 min/sess, 16 wk, 60–80% of 1RM	↓ CRP, ↑ Adiponectin
Dadrass 2019 [19]	Iran	12 (12/0) pts 54.91 ± 5.86 y	12 (12/0) pts 53.16 ± 8.12 y	3 sess/wk, 50 min/sess, 12 wk, 55–75% of 1RM	↔ CRP, ↓ IL-6, ↓ TNF-α
Ghodrat 2022 [20]	Iran	7 (0/7) pts 45–65 y	8 (0/8) pts 45–65 y	3 sess/wk, 40–70% 1RM	↔ CRP
Jangjo-Borazjani 2021 [21]	Iran	10 (0/10) pts 44.13 ± 1.19 y	10 (0/10) pts 42.9 ± 3.2 y	3 sess/wk, 45 min/sess, 8 wk, 60–80% of 1RM	↓ CRP
Jorge 2011 [22]	Brazil	12 (5/7) pts 54.10 ± 8.94 y	12 (4/8) pts 53.42 ± 9.82 y	3 sess/wk, 60 min/sess, 12 wk	↓ hsCRP, ↔ IL-6, ↔ TNF-α, ↔ Adiponectin, ↔ Resistin
Kadoglou 2012 [23]	Greece	23 (7/16) pts 61.5 ± 5.4 y	24 (5/19) pts 64.6 ± 4.3 y	3 sess/wk, 45–60 min/sess, 12 wk, 60–80% of 1RM	↔ hsCRP
Kadoglou 2013 [5]	Greece	23 (7/16) pts 56.1 ± 5.3 y	24 (7/17) pts 57.9 ± 7.2 y	4 sess/wk, 60 min/sess, 24 wk, 60–75% HRmax	↔ hsCRP
Ku 2010 [24]	Korea	13 (0/13) pts 55.7 ± 6.2 y	16 (0/16) pts 57.8 ± 8.1 y	5 sess/wk, 12 wk, 40–50% VO2max	↔ Adiponectin
Magalhaes JP 2020 [25]	Portugal	25 (15/10) pts 56.7 ± 8.3 y	27 (14/13) pts 59.0 ± 8.1 y	3 sess/wk, 52 wk	↔ CRP, ↓ IL-6, ↔ TNF-α
Mavros Y 2014 [26]	Australia	30 pts ^† ‡^	39 pts ^† ‡^	3 sess/wk, 52 wk, 80% of 1RM	↔ CRP
Miller 2017 [27]	Australia	16 (10/6) pts 67.6 ± 5.2 y	13 (6/7) pts 66.9 ± 5.3 y	3 sess/wk, 45 min/sess, 26 wk, 75–85% of 1RM	↔ hsCRP, ↔ IL-6, ↔ TNF-α, ↔ Adiponectin, ↔ Resistin
Rech 2019 [28]	Brazil	17 (10/7) pts 70.5 ± 7.4 y	21 (10/11) pts 68 ± 6.5 y	3 sess/wk, 12 wk	↔ CRP, ↔ IL-6, ↔ TNF-α
Sabouri M 2021 [29]	Iran	15 (7/8) pts 51.31 ± 4.47 y	13 (6/7) pts 52.28 ± 3.16 y	3 sess/wk, 12 wk, 100% of 1RM	↓ CRP, ↓ IL-6, ↔ TNF-α
Swift 2012 [30]	USA	58 (26/32) pts 58.7 ± 8 y	37 (11/26) pts 58.5 ± 8.6 y	3 sess/wk, 39 wk	↔ CRP
Wycherley TP 2010 [31]	Australia	17 ^† ‡^	16 ^† ‡^	3 sess/wk, 45 min/sess, 16 wk, 70–85% of 1RM	↔ CRP

Footnotes: ^†^ age not reported; ^‡^ gender distribution not reported. Post-intervention changes explanation: ↔ no statistically significant change, ↓ statistically significant reduction, ↑ statistically significant increase. Abbreviations: pts, patients; y, year; sess, sessions; wk, week; min, minutes; 1RM, 1-repetition maximum; HRmax, heart rate maximum; VO2max, maximum exercise capacity; hsCRP, high sensitivity C-reactive protein; CRP, C-reactive protein; IL-6, interleukin-6; TNF-α, tumor necrosis factor-a.

**Table 3:** Assessment of risk of bias of the randomized control trials included in the meta-analysis.

	Random Sequence Generation	Allocation Concealment	Blinding of Participants and Personnel	Blinding of Outcome Assessment	Incomplete Outcome Data	Selective Reporting	Other Bias
Bhati 2023 [17]							
Brooks 2007 [18]							
Dadrass 2019 [19]							
Ghodrat 2022 [20]							
Jangjo-Borazjani 2021 [21]							
Jorge 2011 [22]							
Kadoglou 2012 [23]							
Kadoglou 2013 [5]							
Ku 2010 [24]							
Magalhaes JP 2020 [25]							
Mavros Y 2014 [26]							
Miller 2017 [27]							
Rech 2019 [28]							
Sabouri M 2021 [29]							
Swift 2012 [30]							
Wycherley TP 2010 [31]							

Footnotes: green = low risk, yellow = unclear risk, red = high risk.

## Data Availability

The original contributions presented in this study are included in the article. Further inquiries can be directed to the corresponding author.
